# Fostering EFL learners’ motivation, anxiety, and self-efficacy through computer-assisted language learning- and mobile-assisted language learning-based instructions

**DOI:** 10.3389/fpsyg.2022.899557

**Published:** 2022-08-12

**Authors:** Li Dong, Shireen Jamal Mohammed, Khaled Ahmed Abdel-Al Ibrahim, Afsheen Rezai

**Affiliations:** ^1^Linguistics and Applied Linguistics, College of Liberal Arts, Anhui Normal University, Wuhu, China; ^2^Department of Foreign Languages, Wannan Medical College, Wuhu, China; ^3^Department of Translation, College of Languages, Nawroz University, Duhok, Iraq; ^4^Department of Educational Psychology, College of Education, Prince Sattam Bin Abdulaziz University, Al-Kharj, Saudi Arabia; ^5^Department of Psychological Education, Sohag University, Sohag, Egypt; ^6^Teaching English and Linguistics Department, University of Ayatollah Ozma Borujerdi, Borujerd, Iran

**Keywords:** mobile-assisted language learning (MALL), computer-assisted language learning (CALL), foreign language learning motivation, foreign language anxiety, self-efficacy

## Abstract

In the literature, a mass of studies have inspected the effects of computer-assisted language learning (CALL) and mobile-assisted language learning (MALL) on Iranian English as a Foreign Language (EFL) learners’ achievement. However, the effects of CALL and MALL on psychological factors, such as motivation, anxiety, and self-efficacy, have largely remained unexplored. Thus, this study explored the effects of CALL and MALL, and face-to-face (FTF) learning environments on Iranian EFL learners’ motivation, anxiety, and self-efficacy. To this aim, using a random sampling method, a total of 137 male EFL intermediate learners were selected and homogenized using the Oxford Quick Placement Test (OQPT). Based on the test scores, a total of 90 EFL learners were selected and randomly assigned to three groups, namely, CALL (*n* = 30), MALL (*n* = 30), and FTF (*n* = 30). Then, the participants’ motivation, anxiety, and self-efficacy were gauged prior to the instructions. Afterward, they received CALL-based, MALL-based, and conventional instructions which lasted 25 1-h sessions held twice a week. At the end of the instructions, the participants’ motivation, anxiety, and self-efficacy were measured again. The collected data were analyzed through a one-way MANOVA. Findings evidenced that the experimental groups’ motivation, anxiety, and self-efficacy were positively affected by the CALL-based and MALL-based instructions. However, there was not a statistically significant difference between the CALL group and MALL group concerning the gains of motivation, anxiety, and self-efficacy. In light of the findings, a range of implications is suggested for relevant stakeholders.

## Introduction

It is deemed that new technologies have an undeniable role in our daily and academic life. They have been adopted as an inseparable part of life and the means of everyday communication (Garrett, 2009). Young generations are known as the digital natives (Bennett et al., 2008) since the new technologies are among the first things they face and experience in their surrounding environments (Naseri and Motallebzadeh, 2016). As Ghobadi and Taki (2018) note, nowadays, students are more involved in and constantly connected to the net to seek new information. As students prefer independent learning styles, they have a high inclination to use new technologies for promoting their learning (Rahimi and Yadollahi, 2011; Azizi et al., 2022a).

Using the new technologies in second language (L2) education is widely recognized, including online methods, systems, instruments, techniques, and new materials to make the way for L2 learners to achieve their intended educational objectives (Hazaea and Alzubi, 2016). The new technologies offer some outstanding advantages, such as easy access in preparing and delivering the contents to L2 learners (Obari and Lambacher, 2015). However, it should be underscored that applying the new technologies in teaching and learning English should integrate novel tools and resources (Ertmer, 2005; Alemi et al., 2015).

One of the staple applications of the new technologies in L2 education is computer-assisted language learning (CALL). As Tomlinson (2012) notes, CALL materials are accessible on websites, computers, courseware, and online courses not to be confused with Information Communication Technology (ICT). He defines ICT materials as the applications utilized for conveying the materials and helping interactions, and other web sources, such as YouTube and social media. Some outstanding merits have been listed for CALL, including (1) making teaching and learning interesting; (2) granting learners opportunities to take their learning responsibility; (3) giving learners active roles in the learning processes; and (4) offering learners imaginative things that can be displayed *via* computer simulations (Dina and Ciornei, 2013; Azizi, 2022). Due to such noticeable merits, it can simplify and facilitate learning processes for English as a Foreign Language (EFL) learners (; Beatty, 2013; Vadivu and Chupradit, 2020). It is interesting to note that the previous studies have documented that the learners trained *via* the CALL-based programs gained more promising results compared to the conventional teaching methods (Nim Park and Son, 2009; Nachoua, 2012; Tafazoli et al., 2020).

In addition to computer devices, mobile devices and technologies have increasingly been welcomed and applied to realize educational objectives (Pettit and Kukulska-Hulme, 2007; Burston and Giannakou, 2022). Its users, both instructors and learners, are getting accustomed to using them to make their instructions as worldwide as possible (Ling and Donner, 2009; Xu and Peng, 2017; Li and Lan, 2022). Further, the advent and expansion of the net have made distance and open learning an opportunity for all people to receive instruction from all parts of the globe (Ratnaningsih et al., 2019). After a while, the attractiveness of distance and open instruction has supported the benefits of mobile devices to be considered as beneficial tools to realize educational purposes (Yang, 2013; Puebla et al., 2022). Following this trend, many scholars have made increasing attempts to make mobile devices a rich learning source (Oberg and Daniels, 2012; Yurdagul and Oz, 2018). Pachler et al. (2010) opine that mobile-assisted language learning (MALL) is concerned with using mobile technologies in L2 education. MALL is the incorporation of mobile devices into L2 learning and teaching (Ahmadi, 2018; Li, 2022). To put it simply, it is the use of mobile technologies to facilitate L2 learning.

A crucial dimension of CALL- and MALL-based instruction is related to the affective factors which immensely affect L2 learners’ achievement (Bodnar et al., 2016). These affective factors, such as motivation, anxiety, and self-efficacy are complex and multi-faceted concepts that, as Dornyei and Ushioda (2011) note, are “responsible for why people decide to do something, how long they are willing to sustain the activity, (and) how hard they are going to pursue it” (p. 4). It is clear that L2 learners’ actual performance and final learning achievements are highly affected by their motivation, anxiety, and self-efficacy. Thus, to gain a credible understanding of the effects of CALL- and MALL-based instruction, L2 learners’ motivation, anxiety, and self-efficacy should be taken into account.

As the use of CALL and MALL may affect L2 learners cognitively, affectively, and bodily, it is essential to explore if they affect EFL learners’ psychological factors. However, a quick glance at the past literature reveals that the effects of CALL and MALL on Iranian EFL learners’ psychological factors, such as motivation, anxiety, and self-efficacy have remained largely unexplored. In response to this long-lasting gap, the present study aimed to explore the effects of CALL and MALL on Iranian EFL learners’ motivation, anxiety, and self-efficacy. The results of the present study may be helpful for EFL teachers to deliver the learning materials such that they can increase L2 learners’ motivation and self-efficacy, and decrease their anxiety. Additionally, it is hoped that the results of this study can further pertinent stakeholders’ understanding of the significance of psychological factors in L2 learning in online classes. As such, they may be in a better position to raise EFL learners’ motivation and self-efficacy and decrease their anxiety. Finally, the results of the present study can enrich the literature of CALL and MALL and open up new avenues for further research in the future.

## Review of literature

### Computer-assisted language learning and mobile-assisted language learning

The enormous development of new information technologies and communication has made drastic changes in the educational systems over recent decades (Bashori et al., 2020; Rahimi et al., 2021). Applying CALL has increased vastly and impacted substantially educational improvements (Hanafiah et al., 2022). CALL is one of the promising methods that have a strong effect on boosting EFL learners’ competence (Tafazoli et al., 2020). This issue of whether CALL is useful to improve L2 learning has received noticeable attention across the world (Rahimi and Yadollahi, 2011; Beatty, 2013). The majority of the studies have verified the valuable roles of CALL in enhancing L2 learning (Nim Park and Son, 2009; Pirasteh, 2014). CALL has been utilized for several different purposes, such as practicing, performing drills, teaching methods, and even making discussion (Garrett, 2009). However, as Crossman (1997) stresses, L2 teachers usually have challenges using CALL efficiently.

Levy (1997) defines CALL as “using and studying the applications of the computers in teaching and learning a language” (p. 1). One of the noticeable advantages of CALL is that it allows L2 teachers and L2 learners to teach and learn at their own pace (Nachoua, 2012). According to Tatiana Dina and Ciornei (2013), CALL has can facilitate interactions in online classes. It offers learning practices in various forms, offers constructive feedback on students’ performance, encourages group and pair works, boosts EFL learners’ self-regulated learning, paves the way for to reach the different resources, facilitates effective interactions, individualizes instructions, and motivates EFL learners (Beatty, 2013; Shadiev and Yu, 2022).

The other application of technology in L2 education includes MALL. Mobile devices have become an indispensable component of our everyday lives (Lindaman and Nolan, 2015). They have immensely affected our lifestyles in general, and our learning styles, in particular (Viberg et al., 2020). They can provide abundant effective uses in L2 education (Ebadijalal and Yousofi, 2021). In this regard, rather than stopping L2 learners from applying their smartphones in the classrooms, L2 teachers may want to find ways to accommodate and prepare them for real-world learning experiences in and outside of the classrooms (Ahmed and Ganapathy, 2021; Bashori et al., 2021).

According to Xu and Peng (2017), MALL is defined as using mobile tools to accelerate L2 learning and teaching. L2 learners do not always have to study in real classrooms, but they may have the opportunities to learn through mobile instruments (Hsu, 2013). In other words, MALL can move L2 teachers and L2 learners out of the classrooms into the authentic world. Through mobile technologies, L2 teachers can create a rich learning environment (Yang, 2013). MALL involves using any moveable learning resources; therefore, it encompasses audio cassettes, books, audio CDs, DVD players, and portable radios (Derakhshan, 2011; Azara and Nasiri, 2014; Chupradit et al., 2020). The possibility of learning English *via* mobile devices without the limitations of time and place substantially increases L2 learners’ motivation because they feel more responsible for their own learning (Kukulska-Hulme and Shield, 2008; Lindaman and Nolan, 2015). This, accordingly, can make them have control over the learning processes (Vadivu and Chupradit, 2020).

Previous studies (e.g., Yurdagul and Oz, 2018; Dağdeler et al., 2020), have supported the effectiveness of MALL in cultivating L2 learners’ achievement. Of particular note is that, as Kukulska-Hulme (2005) stresses, MALL differs from CALL in its use of personal, portable tools since it enables novel learning methods and offers spontaneity or continuity of accesses and interactions across different levels of use. From this viewpoint, MALL is different from CALL as it is more learner-centered. Additionally, as mobile devices are cheaper than computers, they are used by most of the students nowadays and are considered as an integral part of daily life. As noted by Chinnery (2006), though computers are better at handling a large amount of information, mobile devices are superior in terms of portability. Further, one of the clear distinctions between MALL and CALL is that mobile devices present more efficient ways of learning by focusing on spontaneity, continuity, and privacy (Chaka, 2009).

### Motivation, anxiety, and self-efficacy in second language learning

One of the common psychological factors affecting L2 learning is motivation. It accounts for “why individuals make a decision to perform something, how long they are going to keep the activities, and how difficult they are willing to follow it (Dornyei, 2001, p. 8). Ryan and Deci (2000) note that “to be motivated implies persuading to conduct a task or an activity” (p. 54). Contrary to the unmotivated individuals who lose propulsion and inspiration to do a task, motivated individuals are energetic to do it well. Curiosity, inclination, interest, or a desire to reach intended goals are the fundamental agents, composing motivated individuals (Williams and Burden, 1997). Nonetheless, it should be noted that arousing interest is not adequate to be inspired, but it must be kept as well. In addition, energy and time must be invested, and the required effect needs to be maintained to achieve the desired goal (Steers and Porter, 1991; MacIntyre and Vincze, 2017; Seneviratne et al., 2019).

The crucial role of motivation in developing L2 learning is indisputable. Lifrieri (2005) affirms that when it is asked about the factors affecting levels of success, most individuals point to motivation. According to Brown (2000), L2 learners with high motivation become more successful. In the same vein, Gardner (2006) asserts that L2 learners with higher motivation understand better than L2 learners with low motivation. If an L2 learner is motivated, they have reasons for involving in the given activities, making more efforts, persisting in the tasks, focusing on the activities, showing desires to reach the goals, and enjoying learning (Oxford and Shearin, 1994; Oroujlou and Vahedi, 2011; MacIntyre and Vincze, 2017). In relation to online learning, the success of L2 learners largely relies on their abilities to be actively engaged with the digital resources, as well as initiate and sustain meaningful communications with other users (Moè, 2016; Jones, 2020). To these ends, L2 learners’ motivation, self-regulation learning, and positive learning dispositions are of critical importance (Salmee and Arif, 2019; Moè and Katz, 2020).

A theory presented as a theoretical framework for this study is Self-Determination Theory, developed by Deci and Ryan (Deci and Ryan, 1985). It is predicated on the assumption that humans’ motivation to perform a task is determined by three basic psychological needs: competence, autonomy, and relatedness (Gagné and Deci, 2005; Jeno et al., 2017). As Ryan et al. (2006) note, when individuals’ needs of competence, autonomy, and relatedness are fulfilled, they become self-determined to accomplish a task. SDT has provided strong explanations for students’ motivation and engagement in online classes (Ryan et al., 2006; Przybylski et al., 2009; Tamborini et al., 2010; Huang et al., 2019).

Anxiety is another psychological factor affecting L2 learners’ achievement. It is a psychological concept, generally considered as a state of apprehension, an ambiguous fright that is only indirectly concerned with objects (Scovel, 1991). As perceived intuitively by L2 learners, it adversely affects L2 learning (Horwitz, 2001). According to Brown (2000), there are three kinds of anxiety: trait anxiety, state anxiety, and situation-specific anxiety. Trait anxiety refers to the global or general anxiety and students’ constant feelings of anxiety in different situations. State anxiety refers to a relatively fixed disposition based on which the individuals judge a wide range of situational events as naturally threatening (Brown, 2000). State anxiety refers to feelings of stress and fear that L2 learners experience when facing threats. It is temporary anxiety, a response to a stimulus that causes anxiety. The situation-specific anxiety is a type of anxiety in which the students are anxious in particular contexts.

The other psychological factor influencing L2 learners’ achievement is self-efficacy (Pajares, 2006; Kim and Shin, 2021). It is defined as students’ beliefs in their capabilities to succeed in doing tasks (Bernhardt, 1997). It influences individuals’ decisions, attempts, and behaviors in difficulties and challenges (Bandura, 1986; Esmaili et al., 2021). It also affects the levels of anxiety that L2 learners experience while doing tasks. Accordingly, the way students select their behaviors is influenced by self-efficacy. In actual fact, it is a stronger predictor of success or failure than other psychological factors (Sun et al., 2021). L2 learners with higher self-efficacy make more efforts in doing the required tasks and are more tenacious (Bandura, 1986; Pajares, 2000; Azizi et al., 2022b; Xu et al., 2022). Self-efficacy can affect L2 learners’ emotions. Encountering challenges, L2 learners with low self-efficacy may consider situations more demanding and more complicated than they are (Alharbi, 2021). This can result in greater anxiety and stress levels among L2 learners and may make them demotivated. Bandura (1997) points to four origins of self-efficacy: (1) mastery experiences (i.e., our achievement raises our levels of self-efficacy); (2) vicarious experiences (i.e., other students’ achievement motivates the rest to believe that they have the same abilities in achieving fruitful results); (3) persuasions (i.e., what others state influences our beliefs about our capabilities); and (4) psychological conditions (i.e., stress, fear, and anxiety affect our behaviors).

#### Effects of computer-assisted language learning and mobile-assisted language learning on second language learning

Considering the effects of CALL and MALL on L2 learning, a range of empirical studies has been conducted in the literature. In a study, Khoshsima and Mozakka (2017) examined the impacts of CALL on EFL learners’ listening comprehension. Their findings demonstrated that using CALL led to significant development in the learners’ listening comprehension. Besides, Alotumi (2018) investigated the effects of CALL on Yemeni EFL learners’ score attainment on the TOEFL iBT test. The results evidenced that there were significant differences between the CALL group and the conventional group concerning the gains on the TOEFL iBT test. In addition, Grenner (2019) reviewed the previous studies to disclose how CALL might encourage L2 learners to enhance their learning. The results disclosed that CALL as a motivational method could lead to promising outcomes through supplying authentic materials and creating learner-centered environments. Moreover, Shafiee et al. (2019) scrutinized the effects of CALL-based and Non-CALL-based instructions on Iranian EFL learners’ reading comprehension. They found that the CALL group did outweigh the non-CALL group on the reading comprehension post-test. Further, Dağdeler et al. (2020) investigated the influences of MALL on EFL students’ collocation learning. Their results evidenced that there was a significant difference between the experimental group and the control group in terms of gains of collocation knowledge-building. Additionally, Jamshidi and Zenouzagh (2020) explored the effects of MALL on Iranian EFL students’ reading comprehension. The results indicated that the experimental group outperformed the control group regarding the gains of the reading comprehension. Plus, Namaziandost et al. (2021) investigated the impact of the CALL-based Rosetta Stone application and the Mall-based Rosetta Stone application on Iranian EFL learners’ vocabulary development. They found that the experimental groups significantly outflanked the control group at the end of the interventions. Finally, Hanafiah et al. (2022) inspected the effects of CALL on Indonesian EFL learners’ vocabulary learning, speaking skill, and speaking anxiety. Their results indicated that CALL positively affected the participants’ vocabulary learning, speaking skill, and speaking anxiety.

Concerning the psychological factor, the effects of robot-assisted language learning (RALL) on relieving Iranian high school students’ anxiety in L2 vocabulary learning were investigated by Alemi et al. (2015). The experimental group was trained by an English teacher accompanied by a humanoid robot assistant. The results uncovered that the experimental group could relieve their anxiety better at the end of the treatments. Further, recently Nasri et al. (2021) explored the effects of CALL-based instruction on Iranian EFL learners’ motivation and attitudes. Their findings documented that the experimental group’s motivation in L2 learning significantly improved compared to the control group. Further, their results showed that the participants trained through CALL shaped positive attitudes toward L2 learning.

As it may be implied from the above-reviewed studies, they have addressed the effects of CALL and MALL on the learning of language components (e.g., grammar and vocabulary) and language skills (e.g., listening, speaking, reading, and writing). However, the effects of CALL and MALL on the psychological factors have received scant attention in the EFL context of Iran. Therefore, the present study aimed to fill in the gap by disclosing the effects of CALL and MALL on Iranian EFL learners’ motivation, anxiety, and self-efficacy. To meet these objectives, the following research question was put forward:

**RQ.** Does applying CALL and MALL have any positive effects on Iranian EFL learners’ motivation, anxiety, and self-efficacy?

In line with the research question above, the null hypothesis below was investigated:

**H0.** Applying CALL and MALL does not have any positive effects on Iranian EFL learners’ motivation, anxiety, and self-efficacy.

## Method of the study

### Design

To run the present study, the researchers used a true-experimental design. After homogenizing 137 pre-intermediate EFL learners, a total of 90 students whose language proficiencies were the same were selected and randomly assigned to three groups, namely CALL, MALL, and face-to-face (FTF) groups. Then, they went through pre-test, interventions, and post-test procedures. In sum, to explore the effects of CALL, MALL, and FTF environments on Iranian EFL learners’ motivation, anxiety, and self-efficacy, the researchers implemented a true-experimental design.

### Participants

The present study was run at Iran Language Institute (ILI) in Borujerd, Iran. The researchers selected 137 intermediate EFL learners using a random sampling method. According to Riazi (2016), the random sampling method is used to grant an equal chance to all the individuals in a population to participate in a study. As the education is run based on gender-segregation policy in Iran, the participants included just male students who aged from 16 to 32 years old. The primary reason to select the participants was the easy availability to the researchers. Based on the principal’s report of ILI, the participants had taken rigorous tests, and based on their performance, they had been ranked as intermediate. However, to assure that the participants were at the same level of language proficiency, they became homogenized through the Oxford Quick Placement Test (OQPT). The participants whose scores fell 1 SD above and 1 SD below the mean score were selected. In total, 90 EFL participants regardless of their ages were selected and randomly assigned to three groups, namely CALL, MALL, and FTF groups. Of particular note is that the participants were learning English as a foreign language and they did not have opportunities to learn English outside the walls of the institute. They were learning English for 4 h per week. It is worth noting that the participants expressed their consent to participate in the study orally and the researchers said that they could withdraw from the study as they wished. More importantly, the researchers ensured that the participants’ performances during the study would remain confidential and they would inform them about the final results. It should be noted that the researchers recruited three EFL teachers, holding M.A. in TEFL to run the instructions for the three groups.

### Instruments

The researchers used four instruments to gather the needed data. The first instrument included the OQPT used to make the participants homogenized. The major reason for using this test was that the researchers consulted two university professors in Applied Linguistics and they confirmed that it could meet the purposes of the study. The OQPT test comprises one hundred multiple-choice items, measuring L2 learners’ vocabulary, grammar, and reading comprehension abilities. It entails 40 vocabulary items, 40 grammar items, and 20 reading comprehension items. It should be noted that the participants whose scores fell around the mean score were selected for the main study.

The second tool was Attitude/Motivation Test Battery (AMTB), designed and validated by Gardner (2004). It was used to measure the participants’ motivation and attitude level to learn English. AMTB consists of 26 items, measuring three important factors, including motivational intensity, desire to learn English, and orientation index. It comprises five-point Likert scale items ranging from 1 (strongly disagree) to 5 (strongly agree).

The third instrument was the Foreign Language Classroom Anxiety Scale (FLCAS), designed and validated by Horwitz et al. (1986). FLCAS deals with the fear of L2 in a course, such as the fear of speaking in front of other students. It includes 33 items. For instance, item 12 is “I do not worry about making mistakes in language class.” It comprises five-point Likert scale items, ranging from 1 (strongly disagree) to 5 (strongly agree).

The last instrument included the self-efficacy questionnaire, designed and validated by Ghonsooly and Elahi (2008). The questionnaire was used to measure the participants’ level of self-efficacy in learning English. It contains 14 items in a Likert-scale format ranging from 1 (strongly disagree) to 5 (strongly agree).

Of particular note is that the researchers invited two experts in translation to translate the questionnaires into the participants’ mother tongue (Persian). The reason for this was to increase the validity of the responses by avoiding any probable misunderstanding on the part of the participants. It should be underscored that the reliability and validity of the instruments were measured through a pilot study. They were administered to 20 EFL learners who were similar to participants in terms of gender, age, and language proficiency at another private language institute. According to Riazi (2016), the sample was large enough to assess the reliability and validity of the instruments. The calculated reliability for OQPT was (*r* = 0.92), for AMTB was (*r* = 0.81), for FLCAS was (*r* = 0.83), and for the self-efficacy questionnaire was (*r* = 0.87), respectively. Regarding the validity, the researchers invited two university professors in Applied Linguistics to assess if they were appropriate for the current study in terms of face and content. In general, they confirmed that they were appropriate fits for the objectives of the present study.

### Data collection procedures

To run the present study, the researchers took some steps, in order. At the first step, they recruited two experts in translation to translate the questionnaires into the participants’ mother tongue (Persian). At the second step, they run a pilot study to assess the reliability and validity of the instruments. At the third step, they administered the OQPT test to homogenize the participants. The students whose scores fell around the mean score (*n* = 90) were selected and randomly assigned to three groups, namely CALL, MALL, and FTF groups. At the fourth step, they implemented the questionnaires to measure the learners’ motivation, anxiety, and self-efficacy prior to the treatments. At the fifth step, the treatments were run for the groups. Prior to running the instructions, the researchers held a mini-workshop with the EFL teachers to inform them about the objectives of the study and rest assured if they knew how to run the classes in the different learning environments It should be noted that the instructional materials used to run the classes included three units of Four Corners level 2 (Richards and Bohlke, 2011). Every unit includes different tasks to cultivate L2 learners’ communicative competence. It is worth noting that the CALL group received the instructions *via* computers and the MALL group received the instruction *via* smartphones. That is, the researchers assured that the different groups received the instructions *via* computers and smartphones.

For the CALL group, the instruction was offered through a Skype program. The researchers ensured that the participants received the instruction at home. It is a free computer program that allows users to make telephone calls over the internet, to make conference calls and video calls, to chat, and to transfer files to teach the participants’. In each session, one part of the textbook was taught to the participants online, and the teacher and learners worked in a simultaneous learning setting. The participants could chat and discuss the materials online, and everything was carried out on an online platform. In this virtual setting, the teacher used different learning materials like pictures and short movies to facilitate the learning. The learners could freely join the class, share their opinions, and raise their questions. Additionally, they were capable of joining or leaving the classroom without any limitation. The MALL group was trained through a MALL-based instruction; that is through WhatsApp application. They received the instruction at home. This application was used since it was accessible to all the participants, easy to use, and free. The researchers established a group for the learners and invited them to join it. Once each part was sent to the group, the teacher explained its content and read out the task. The students were allowed to post their respective questions on the group page after each conversation and reading text had been explained. The required feedback on the learners’ performance and assignments were sent *via* messages or audio formats to the group. The participants could raise their questions and offer feedback on their peers’ performance. More importantly, the teachers could share learning materials with different formats, such as audio and video. The FTF group was trained using a traditional method. They attended an FTF class at the institute, and the teacher taught one conversation to them in each session; after teaching ten conversations, the teacher taught them ten reading texts (one reading in each session). Having completed the interventions, the researchers administered the questionnaires to measure the participants’ motivation, anxiety, and self-efficacy.

### Data analysis procedures

The researchers used SPSS, version 22 to analyze the collected data. In addition to calculating the basic descriptive statistics, such as mean (M) and standard deviation (SD), the researchers run a one-way MANOVA and *Post hoc* Scheffe to determine the effects of the different environmental learnings on the participants’ motivation, anxiety, and self-efficacy. The one-way MANOVA, as noted by Riazi (2016), is a statistical procedure to disclose if there are any differences between independent groups and more than one continuous dependent variable.

## Results

As noted above, the researchers used a one-way MANOVA to analyze the collected data. Before running it, the researchers checked out if its assumptions were met. They checked out the linearity assumption and the distribution of scores for each of the groups on the scatterplot matrix. They did not observe any curvilinear relationship. Besides, they checked out the normality assumption through a Kolmogorov–Smirnov test. As the Sig. values (0.25) were larger than the critical value (0.05), they concluded that the data were normally distributed. Having assured that the required assumptions were met, they employed a one-way MANOVA. As reported in Table 1, this study included two categorical, independent variables with three levels, namely CALL, MALL, and FTF. Each group included 30 participants.

**TABLE 1:** Results of descriptive statistics.

	Groups	*M*	SD	*N*
Anxpost	CALL	133.0667	10.29876	30
	MALL	134.4667	9.52215	30
	FTF	84.0333	30.57662	30
	Total	117.1889	30.41211	90
MotPost	CALL	106.9667	11.60999	30
	MALL	108.9000	10.34025	30
	FTF	59.1667	14.07635	30
	Total	91.6778	26.04569	90
SelfPost	CALL	56.3667	9.23816	30
	MALL	57.6000	8.95044	30
	FTF	32.9333	8.79629	30
	Total	48.9667	14.46922	90

As presented in Table 2, a Wilk’s lambda value of 0.081 with a significant value of 0.00 < 0.05 was obtained. Therefore, among the three groups, there existed a statistically significant difference regarding anxiety, motivation, and self-efficacy.

**TABLE 2:** Results of multivariate tests.

Effect		Value	*F*	Hypothesis df	Error df	Sig.	Partial eta squared
Intercept	Pillai’s trace	0.670	55.576	3.000	82.000	0.000	0.670
	Wilks’ lambda	0.330	55.576	3.000	82.000	0.000	0.670
	Hotelling’s trace	2.033	55.576	3.000	82.000	0.000	0.670
	Roy’s largest root	2.033	55.576	3.000	82.000	0.000	0.670
Anxpre	Pillai’s trace	0.053	1.534	3.000	82.000	0.212	0.053
	Wilks’ lambda	0.947	1.534	3.000	82.000	0.212	0.053
	Hotelling’s trace	0.056	1.534	3.000	82.000	0.212	0.053
	Roy’s largest root	0.056	1.534	3.000	82.000	0.212	0.053
MotPre	Pillai’s trace	0.256	9.392	3.000	82.000	0.000	0.256
	Wilks’ lambda	0.744	9.392	3.000	82.000	0.000	0.256
	Hotelling’s trace	0.344	9.392	3.000	82.000	0.000	0.256
	Roy’s largest root	0.344	9.392	3.000	82.000	0.000	0.256
SelfPre	Pillai’s trace	0.183	6.119	3.000	82.000	0.001	0.183
	Wilks’ lambda	0.817	6.119	3.000	82.000	0.001	0.183
	Hotelling’s trace	0.224	6.119	3.000	82.000	0.001	0.183
	Roy’s largest root	0.224	6.119	3.000	82.000	0.001	0.183
Groups	Pillai’s trace	0.924	23.736	6.000	166.000	0.000	0.462
	Wilks’ lambda	0.081	68.928	6.000	164.000	0.000	0.716
	Hotelling’s trace	11.351	153.243	6.000	162.000	0.000	0.850
	Roy’s largest root	11.347	313.929	3.000	83.000	0.000	0.919

As reported in Table 3, the equality of variances assumption was met for the motivation (*p* = 0.68 > 0.05) and the self-efficacy (*p* = 0.75 > 0.05). However, this assumption is violated regarding anxiety (*p* = 0.00 < 0.05). Therefore, a more conservative alpha level for determining significance of this variable is needed in the univariate *F*-test (Pallant, 2007). As suggested by Tabachnick and Fidell (2007), an alpha of 0.025 or 0.01, rather than the conventional 0.05 level should be reported.

**TABLE 3:** Results of Levene’s test of equality of error variances.

	*F*	df1	df2	Sig.
Anxpost	16.522	2	87	0.000
MotPost	0.374	2	87	0.689
SelfPost	0.281	2	87	0.755

As seen in Table 4, three of the dependent variables, anxiety (0.00 < 0.01), motivation (0.00 < 0.01), and self-efficacy (0.00 < 0.01) recorded a significance value. It evidences that there existed a statistically significant difference among the three groups regarding anxiety, motivation, and self-efficacy.

**TABLE 4:** Results tests of between-subjects effects.

Source	Dependent variable	Type III sum of squares	df	Mean square	*F*	Sig.	Partial eta squared
Corrected model	Anxpost	53280.740	5	10656.148	30.829	0.000	0.647
	MotPost	50274.908	5	10054.982	83.619	0.000	0.833
	SelfPost	12843.592	5	2568.718	37.271	0.000	0.689
Intercept	Anxpost	15191.006	1	15191.006	43.948	0.000	0.343
	MotPost	4444.349	1	4444.349	36.960	0.000	0.306
	SelfPost	2160.217	1	2160.217	31.344	0.000	0.272
Anxpre	Anxpost	424.631	1	424.631	1.228	0.271	0.014
	MotPost	163.904	1	163.904	1.363	0.246	0.016
	SelfPost	37.803	1	37.803	0.549	0.461	0.006
MotPre	Anxpost	91.991	1	91.991	0.266	0.607	0.003
	MotPost	1854.162	1	1854.162	15.420	0.000	0.155
	SelfPost	339.633	1	339.633	4.928	0.029	0.055
SelfPre	Anxpost	517.197	1	517.197	1.496	0.225	0.018
	MotPost	619.116	1	619.116	5.149	0.026	0.058
	SelfPost	493.484	1	493.484	7.160	0.009	0.079
Groups	Anxpost	48672.869	2	24336.434	70.407	0.000	0.626
	MotPost	46214.360	2	23107.180	192.164	0.000	0.821
	SelfPost	11766.851	2	5883.425	85.366	0.000	0.670
Error	Anxpost	29035.049	84	345.655			
	MotPost	10100.747	84	120.247			
	SelfPost	5789.308	84	68.920			
Total	Anxpost	1318307.000	90				
	MotPost	816809.000	90				
	SelfPost	234429.000	90				
Corrected total	Anxpost	82315.789	89				
	MotPost	60375.656	89				
	SelfPost	18632.900	89				

Partial eta squares of 0.62, 0.82, and 0.87 for anxiety, motivation, and self-efficacy, respectively, are considered quite large effect sizes (Tabachnick and Fidell, 2007). These values represented the proportion of the variance in the dependent variables of anxiety, motivation, and self-efficacy that could be justified by the effects of the independent variables, group with three levels of the experimental groups of CALL and MALL and FTF. The large effect sizes documented that 62 percent of the variance in anxiety, 82 percent of the variance in motivation, and 87 percent of the variance in self-efficacy can be ascribed to the effects of the independent variable.

Although the experimental groups of CALL and MALL and FTF differed in terms of anxiety, motivation, and self-efficacy, it cannot be derived from Table 4 that which group had the higher scores. As presented in Table 5, the mean scores for anxiety in CALL and MALL groups (*M*_CALL_ = 132.98, *M*_MALL_ = 134.36) were higher than the mean score of the FTF group (*M* = 82.21). It also shows that the mean scores for motivation in CALL and MALL groups (*M*_CALL_ = 107.22, *M*_MALL_ = 108.27) were higher than the mean score of the FTF group (*M* = 159.82). Furthermore, it shows that the mean score for self-efficacy in CALL and MALL groups (*M*_CALL_ = 56.12, *M*_MALL_ = 57.99) were higher than the mean score of the FTF group (*M* = 32.78).

**TABLE 5:** Results of estimates.

Dependent variable	Groups	Mean	Std. error	95% Confidence interval	
				Lower bound	Upper bound
Anxpost	CALL	132.986	3.397	126.230	139.741
	MALL	134.369	3.406	127.595	141.142
	FTF	84.212	3.401	77.449	90.976
MotPost	CALL	107.220	2.004	103.236	111.205
	MALL	108.271	2.009	104.276	112.266
	FTF	59.542	2.006	55.553	63.531
SelfPost	CALL	56.124	1.517	53.107	59.140
	MALL	57.995	1.521	54.971	61.020
	FTF	32.781	1.519	29.761	35.801

As reported in Table 6, there existed no significant differences between the CALL group and the MALL group concerning their anxiety, motivation, and self-efficacy (*p* = 1 > 0.05). However, there was a statistically significant difference among the FTF group and the CALL group and the MALL group with respect to the anxiety (*p* = 0.00 < 0.05), the motivation (*p* = 0.00 < 0.05), and the self-efficacy (*p* = 0.00 < 0.05).

**TABLE 6:** Results of pairwise comparisons.

Dependent variable	(I) Groups	(J) Groups	Mean difference (I-J)	Std. Error	Sig.	95% Confidence interval for difference	
						Lower bound	Upper bound
Anxpost	CALL	MALL	−1.383	4.817	1.000	−13.150	10.384
		FTF	48.773	4.805	0.000	37.034	60.512
	MALL	CALL	1.383	4.817	1.000	−10.384	13.150
		FTF	50.156	4.824	0.000	38.371	61.942
	FTF	CALL	−48.773	4.805	0.000	−60.512	−37.034
		MALL	−50.156	4.824	0.000	−61.942	−38.371
MotPost	CALL	MALL	−1.051	2.841	1.000	−7.991	5.889
		FTF	47.678	2.834	0.000	40.755	54.602
	MALL	CALL	1.051	2.841	1.000	−5.889	7.991
		FTF	48.729	2.846	0.000	41.778	55.680
	FTF	CALL	−47.678	2.834	0.000	−54.602	−40.755
		MALL	−48.729	2.846	0.000	−55.680	−41.778
SelfPost	CALL	MALL	−1.872	2.151	1.000	−7.126	3.383
		CG	23.343	2.146	0.000	18.101	28.585
	MALL	CALL	1.872	2.151	1.000	−3.383	7.126
		FTF	25.215	2.154	0.000	19.952	30.477
	FTF	CALL	−23.343	2.146	0.000	−28.585	−18.101
		MALL	−25.215	2.154	0.000	−30.477	−19.952

As reported in Table 7, there was a statistically significant difference among the experimental groups (CALL and MALL) and the control group on the combined dependent variables, *F*(6, 164) = 68.99, *p* = 0.00; Wilk’s lambda = 0.081; partial eta squared = 0.71. Considering the results of the dependent variables separately, the anxiety (*F*(2, 84) = 70.40, *p* = 0.00, partial eta squared = 0.62), the motivation (*F*(2, 84) = 192.16, *p* = 0.00, partial eta squared = 0.82), and the self-efficacy (*F*(2,84) = 85.36, *p* = 0.00, partial eta squared = 0.067), there were statistical differences among the three groups. As reported for the mean scores, the CALL group and MALL group gained better results regarding the anxiety (*M*_CALL_ = 132.98, *M*_MALL_ = 134.36), the motivation (*M*_CALL_ = 107.22, *M*_MALL_ = 108.27), and the self-efficacy (*M*_CALL_ = 56.12, *M*_MALL_ = 57.99) compared with the FTF group (*M*_anxiety_ = 82.21, *M*_motivation_ = 159.82, and *M*_*self–efficacy*_ = 32.78). Based on the pairwise comparisons, there were no statistically significant differences between the CALL group and the MALL group regarding the anxiety, motivation, and self-efficacy (*p* = 1 > 0.05). However, there was a statistically significant difference between the FTF group and the CALL group and the MALL group regarding the anxiety (*p* = 0.00 < 0.05), the motivation (*p* = 0.00 < 0.05), and the self-efficacy (*p* = 0.00 < 0.05). Therefore, the null hypothesis was rejected. The results are reported in Table 8.

**TABLE 7:** Results of multivariate tests.

	Value	*F*	Hypothesis df	Error df	Sig.	Partial eta squared
Pillai’s trace	0.924	23.736	6.000	166.000	0.000	0.462
Wilks’ lambda	0.081	68.928	6.000	164.000	0.000	0.716
Hotelling’s trace	11.351	153.243	6.000	162.000	0.000	0.850
Roy’s largest root	11.347	313.929	3.000	83.000	0.000	0.919

**TABLE 8:** Results of univariate tests.

Dependent variable		Sum of squares	df	Mean square	*F*	Sig.	Partial eta squared
Anxpost	Contrast	48672.869	2	24336.434	70.407	0.000	0.626
	Error	29035.049	84	345.655			
MotPost	Contrast	46214.360	2	23107.180	192.164	0.000	0.821
	Error	10100.747	84	120.247			
SelfPost	Contrast	11766.851	2	5883.425	85.366	0.000	0.670
	Error	5789.308	84	68.920			

## Discussion

As noted above, the present research purported to examine the impact of CALL, MALL, and FTF on Iranian EFL learners’ motivation, anxiety, and self-efficacy. The results depicted that the CALL group and the MALL group earned a higher level of motivation, lower level of anxiety, and a higher level of self-efficacy than the FTF group. In fact, the findings of the study indicated that the CALL- and MALL-based instructions could create beneficial learning environments in which the participants got motivated, controlled their anxiety, and increased their self-efficacy. According to the findings of the study, it can be argued that CALL- and MALL-based instructions had the potential to increase the participants’ motivation, lower their anxiety, and boost self-efficacy. That is, since the interventions could involve the learners in real and authentic learning activities and offer them interactive learning experiences, they positively affected the psychological factors.

The findings of the study are in line with those of Khoshsima and Mozakka (2017), revealing that the experimental group who received CALL-based instruction outperformed the control group regarding the gains of the listening comprehension. Besides, the results of the study are congruent with those of Alotumi (2018), reporting that there were remarkable differences between the CALL group and the conventional group regarding the total gain scores and the section gain scores of speaking, reading, writing, and listening. In addition, the findings of the study lend support to the results of Grenner (2019). They disclosed that CALL as a motivational method could result in promoted motivation among the participants by offering authentic materials and creating learner-centered environments. Moreover, the results of the study lend credence to those of Shafiee et al. (2019). They found that the CALL group did outweigh the non-CALL group on the reading comprehension post-test. Further, the findings of this study are consistent with those of Alemi et al. (2015), revealing that the experimental group could relieve their anxiety better due to the positive effects of RALL-based instruction. Finally, The results of the study are in line with those of Nasri et al. (2021), showing that the experimental group’s motivation in L2 learning significantly improved compared to the control group and the participants shaped positive attitudes toward L2 learning after receiving CALL-based instruction.

A line of discussion for the findings of the study can be presented with the help of SDT. Aligned with SDT, it can be argued that since the participants’ psychological needs, including competence, autonomy, and relatedness were fulfilled well in CALL and MALL, they might have become intrinsically motivated to continue learning and further their achievements. This, in turn, might have led to decreasing their anxiety and promoting their self-efficacy. This argument receives support from the previous studies (e.g., Ryan et al., 2006; Przybylski et al., 2009; Tamborini et al., 2010; Huang et al., 2019), revealing that in online classes, when learners’ psychological needs, such as competence, autonomy, and relatedness were fulfilled, they became intrinsically motivated to promote their learning.

To recap the discussion, we can also refer to L2 Motivational Self System model (Dörnyei, 2005, 2009). Aligned with this model, it can be argued that CALL and MALL could create learning environments in which the participants could move toward two important future visions, namely self-guides. That is, due to the positive effects of the instructions, the participants might have achieved the ideal-L2 self to internalize the desired hopes and the ought-to self to feel obligated to become the ideal individual due to the societal responsibilities. This, in turn, might assist the participants to reach an awareness of the discrepancy between desired future self-guides and the perceived plausibility of those self-guides, together with their current experience of L2 learning. These all might have led to increasing their motivation and self-efficacy, as well as decreasing their anxiety.

Another possible explanation for the findings is that CALL and MALL might have led to autonomous learning. That is, along with Namaziandost et al. (2021), it may be argued that through the instructions, the participants might have learned to rely on their abilities to control the learning tasks and obligations in the online classes. Thus, they might have improved their self-efficacy, got motivated to continue learning, and handle their fears. An additional possible explanation for the findings may be ascribed to the fact that the learning materials presented through CALL and MALL were durable. In other words, as Dağdeler et al. (2020) note, since the learning materials in CALL and MALL could remain for an unlimited time, the participants might have had this opportunity to turn back to them, review them, and consolidate their learning. This noticeable advantage might have helped the participants increase their level of motivation and self-efficacy, and, accordingly, control their anxiety.

To discuss the findings of the study, we can also refer to the fact that CALL and MALL were student-oriented instructional methods (Grenner, 2019). They could offer online learning materials to simplify information, sharing outside the limitations of time, and place among the EFL learners. Based on the findings, it may be argued that CALL and MALL could combine self-study with asynchronous interactions to improve learning, and they could be utilized to simplify the learning processes in conventional on-campus instruction, distance education, and continuing education. In other words, CALL and MALL might have granted the learners more freedom to expand their learning processes. They were not confined to time and space. Therefore, it is reasonable to claim that the results of the study could be attributed to this outstanding advantage of CALL and MALL.

To justify the findings of the study, it can also be referred to the online collaborative learning theory, introduced by Harasim (2012). According to this theory, it may be argued that CALL and MALL might have offered the Internet facilities to create collaborative learning settings that might have led to shaping collaboration and knowledge building among the participants. With the presence of oral and written interactions in the virtual environments, the learners might have solved their problems collaboratively *via* the negotiation of meaning and might have constructed the required competencies. Since the participants constructed a good command of English competence, they might have become more motivated, increased their self-efficacy, and handled their anxiety.

A further possible justification for the results of the study is the efficiency of CALL and MALL. Along with the findings, it may be argued that CALL and MALL might have let the teachers deliver the learning materials to the EFL learners more efficiently (Yu, 2019). The teachers could use diverse tools, such as podcasts, videos, pictures, PDFs to facilitate the learners’ learning. By including online resources, the teachers might have been able to extend the lesson plan beyond traditional coursebooks and might have created a learning environment in which the participants found motivating and joyful (Zou and Li, 2015). Moreover, the other justification for the findings may be attributed to the outstanding advantage of CALL and MALL, called cost-effectiveness. Aligned with the findings, it may be argued that CALL and MALL were far more affordable as compared to FTF classes. This might be due to the reality that CALL and MALL might have eliminated the cost points of the students’ commutation to the language institute. Thus, they might have saved their time and money. Besides, it may be argued that the students had access to the course materials online, thus creating a paperless learning environment that might have been more affordable for them.

## Conclusion and implications

As pointed out above, the present research explored the impacts of CALL and MALL on Iranian EFL learners’ motivation, anxiety, and self-efficacy. The findings indicated that using MALL and CALL positively affected the Iranian EFL students’ motivation, anxiety, and self-efficacy. According to the results, it may be concluded that integrating MALL and CALL into L2 education can promote EFL learners’ motivation, decrease their anxiety, and improve their self-efficacy. Applying MALL and CALL may be useful for EFL learners since they can expand learning opportunities outside of the classroom, foster cooperative learning, encourage self-study, and increase self-confidence. To close, since we live in the era of new technological developments, it is clear that L2 education is affected by these non-stop developments. Accordingly, L2 practitioners need to consider online environments as a valuable alternative to make the way for efficient L2 learning.

The findings of this research may deliver some implications to pertinent stakeholders. The first implication is for educational policymakers. They can consider online education as an alternative for conventional education. For this, for example, they can use a blended format where EFL learners can benefit from both online classes and FTT classes. The second implication is for school principals and language institute owners. In order to grant learning opportunities to EFL learners, they need to equip their educational centers with new technologies. The third implication is for teacher educators. They should accommodate online teaching approaches and techniques in their syllabi to make EFL teachers familiar with them. The fourth implication is for materials developers. They need to seek new ways through which the educational materials can be designed using new technologies. The fifth implication is for EFL teachers. They may want to employ MALL and CALL in their classes to help EFL learners overcome their anxiety and increase their motivation and self-efficacy. However, it should be noted that EFL teachers cannot use CALL and MALL efficiently unless they have high digital literacy. Thus, they should give particular attention to promoting it. The final implication is for EFL learners. They should give particular attention to developing their digital literacy to benefit from the new technologies to promote their L2 learning achievements.

A number of suggestions for further research are given considering the limitations imposed on the current research. First, as the present study was run in just one language institute, future studies can be conducted in other language institutes in other parts of the country to promote the external validity of the results. Second, as this study was confined to male EFL learners, further studies are needed to include female EFL learners to give a better picture of the research topic. Third, because the setting of this study was a private language institute, interested researchers can explore the effects of CALL and MALL on EFL learners’ motivation, anxiety, and self-efficacy in other settings, such as high schools and universities. Fourth, since the design of the present study was quantitative, future studies can accommodate qualitative designs, such as interviews and observation to present more credible results. Fifth, as this research was cross-sectional, interested researchers can run a longitudinal study to disclose how EFL learners’ motivation, foreign language anxiety, and self-efficacy change in CALL and MALL over a period of time. Last but not least, as the present study surveyed the effects of CALL and MALL on psychological factors, interested researchers can explore their impacts on EFL learners’ performances.

## Data availability statement

The original contributions presented in this study are included in the article/supplementary material, further inquiries can be directed to the corresponding author.

## Author contributions

All authors listed have made a substantial, direct, and intellectual contribution to the work, and approved it for publication.
